# Melt-Processed Poly(Ether Ether Ketone)/Carbon Nanotubes/Montmorillonite Nanocomposites with Enhanced Mechanical and Thermomechanical Properties

**DOI:** 10.3390/ma12030525

**Published:** 2019-02-10

**Authors:** Ruixue Ma, Bo Zhu, Qianqian Zeng, Pan Wang, Yaming Wang, Chuntai Liu, Changyu Shen

**Affiliations:** Key Laboratory of Materials Processing and Mold, Ministry of Education, National Engineering Research Center for Advanced Polymer Processing Technology, Zhengzhou University, Zhengzhou 450002, China; 18530037928@163.com (R.M.); z971599814@foxmail.com (B.Z.); 15237531293@163.com (Q.Z.); 13298193023@163.com (P.W.); ctliu@zzu.edu.cn (C.L.); shency@zzu.edu.cn (C.S.)

**Keywords:** carbon nanotubes, montmorillonite, poly(ether ether ketone), nanocomposites, mechanical properties, crystallization

## Abstract

The agglomeration problem of nanofillers, for instance, carbon nanotubes (CNTs) in a poly(ether ether ketone) (PEEK) matrix, is still a challenging assignment due to the intrinsic inert nature of PEEK to organic solvents. In this work, organically modified montmorillonite (MMT) was introduced as a second filler for improving the dispersion of CNTs in the PEEK matrix and enhancing the mechanical properties, as well as reducing the cost of the materials. The nanocomposites were fabricated through melt-mixing PEEK with CNTs/MMT hybrids, which were prepared in advance by mixing CNTs with MMT in water. The introduction of MMT improved the dispersion stability of CNTs, as characterized by sedimentation and zeta potential. The CNTs/MMT hybrids were maintained in PEEK nanocomposites as demonstrated by the transmission electron microscope. The mechanical and thermomechanical measurements revealed that CNTs together with MMT had a strong reinforcement effect on the PEEK matrix, especially at high temperature, although it had a negative effect on the toughness. A maximum increase of 48.1% was achieved in storage modulus of PEEK nanocomposites with 0.5 wt% CNTs and 2 wt% MMT at 240 °C, compared to that of neat PEEK. The differential scanning calorimetry results revealed that CNTs accelerated the crystallization of the PEEK matrix while a further addition of MMT played an opposite role. The nucleation activity of the fillers was also evaluated by the Dobreva method.

## 1. Introduction

Poly(ether ether ketone) (PEEK) is a semicrystalline thermoplastic that maintains outstanding mechanical properties with a combination of thermal and chemical resistance [1,2,3,4]. Due to its excellent properties, PEEK is suitable for a wide range of applications from medicine to the automobile and aerospace industries [1,2,3,4,5]. However, PEEK itself does not usually satisfy higher mechanical and thermal requirements under harsh service conditions such as heavy load at extremely high temperature [6,7]. Lots of efforts have been made to further enhance the properties of PEEK via the addition of various fillers [4] such as carbon-fibers [8,9], nano graphitic carbon coated inorganic fullerene-like WS_2_ [10], nano-SiO_2_ [11], montmorillonite (MMT) [12,13], and graphene [14].

Carbon nanotubes (CNTs), which are one of the most important carbon-based nanofillers, have been widely used to reinforce PEEK [4,15,16,17,18,19,20,21,22] and other polymers [23]. However, the dispersion of CNTs in the PEEK matrix is poor [16], due to the intrinsic inert nature of PEEK to organic solvents. In this context, some approaches have been utilized to improve the dispersion of CNTs in PEEK, including using a twin-screw extruder with fractional mixing elements [20], the addition of a compatibilizer or the surface modifications of CNTs [21,22,24]. However, the functionalization introduces defective sites in the nanotubes and severely compromises its electronic properties.

Nanostructured hybrids have also been employed to prepare a new class of polymer nanocomposites as well as to solve the agglomeration problem of nanofillers, for instance, CNTs [25,26,27,28,29,30,31]. The polymer nanocomposites were fabricated through blending polymers with CNTs and MMT directly [27,28,29] or preparing CNTs/MMT hybrids first and then mixing them with polymers [30,31]. The CNTs/MMT hybrids could be synthesized by the growth of CNTs on MMT layers [30,31,32,33]. Recently, Tang et al. proposed a simple approach to obtain the hybrids through mixing CNTs in a MMT aqueous solution [34]. The hybrids revealed an excellent reinforcing effect on poly(ethylene oxide), which is hydrophilic and soluble in water [34]. However, it is not known if the CNTs/MMT hybrids obtained from aqueous solution can be used successfully to reinforce PEEK, which is hydrophobic and especially inert to organic solvents.

In this work, organically modified montmorillonite (MMT) was introduced as a second filler for improving the dispersion of CNTs in the PEEK matrix and enhancing the mechanical properties as well as reducing the cost of the materials. The nanocomposites were fabricated through melt-mixing PEEK with CNTs/MMT hybrids, which were prepared in advance by mixing CNTs with MMT in water. The dispersion stability of CNTs/MMT hybrids in water was characterized by sedimentation and zeta potential, while the morphology of the PEEK nanocomposites was probed by transmission electron microscope (TEM). The mechanical and thermomechanical properties, as well as the crystallization behavior of the nanocomposites, were also investigated. It is shown that the developed nanocomposites reveal much higher storage modulus than the matrix, especially at a high temperature, for instance, 240 °C, which is very important for potential high-temperature applications of PEEK.

## 2. Experimental

### 2.1. Materials

PEEK (150 PF) powder with an average particle size (*D*_50_) of 50 μm was supplied by Victrex (Lancashire, UK). The weight-average molecular weight of the material was about 40,000 g/mol. It had a melting temperature (*T*_m_) of 341 °C, measured by a Q2000 DSC (TA Instruments, Newcastle, UK) upon heating at a rate of 10 °C/min after cooling the sample to 100 °C from the melt at the same rate. Multi-walled carbon nanotubes (CNTs), TNM3, with a purity of > 98 wt%, outer diameter of 10–20 nm, length of 10–30 μm, 0.22 g/cm^3^, and true density of ~2.1 g/cm^3^ were supplied by TimesNano (Chengdu, China). Organically modified montmorillonite (MMT), I.34TCN, with surface modifier concentration of 30–32 wt%, particle size of 14–18 μm, specific gravity of 1.9 g/cm^3^, and bulky density of 250–300 kg/m^3^ was purchased from Nanocor (Chapel Hill, NC, USA). The surface modifier was methyl bis hydroxyethyl octadecyl ammonium.

### 2.2. Materials and Sample Preparation

**Figure 1:**
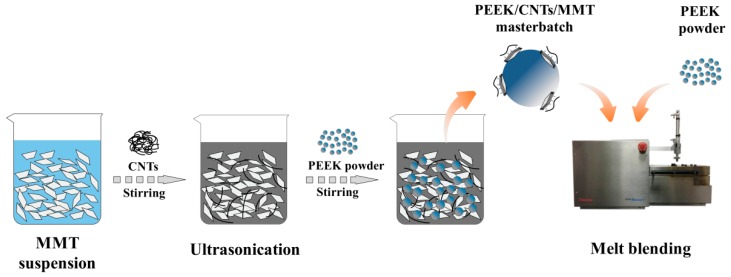
Schematic illustration of the fabrication of multi-walled carbon nanotubes (CNTs)/organically modified montmorillonite (MMT) hybrids and poly(ether ether ketone) (PEEK)/CNTs/MMT nanocomposites.

The schematic diagram for the preparation of PEEK/CNTs/MMT nanocomposites is shown in Figure 1. The procedure includes preparations of CNTs/MMT hybrids and a PEEK/CNTs/MMT masterbatch, and melt-mixing of the masterbatch with neat PEEK. Firstly, MMT was dispersed in deionized water and then added CNTs to MMT suspension with the ratio of 1:0, 1:1, 1:2, and 1:4. It was then stirred with a magnetic stirrer for 10 min, the mixtures were sonicated for 40 min by a Scientz–IID ultrasonic homogenizer (60 W, Scientz Biotechnology, Ningbo, China). The PEEK/CNTs/MMT masterbatch was prepared by adding PEEK powders to the CNTs/MMT suspension and then stirred for 10 min to coat CNTs/MMT hybrids on the surface of PEEK powder, and then filtered it with a 0.22 μm pore size PTFE membrane to remove water. After filtration, the materials were dried in an oven at 150 °C for 3 h under vacuum. For the masterbatch, the content of CNTs was fixed at 5 wt% of the total weight. Finally, the masterbatch was diluted with neat PEEK to fabricate the PEEK/CNTs/MMT nanocomposites by melt-blending with Haake MiniLab II (Thermal Scientific, Waltham, MA, USA) at 380 °C and 50 rpm for 10 min. In the final PEEK/CNTs/MMT nanocomposites, the concentration of CNTs was fixed at 0.5 wt% of the total weight, as shown in Table 1. Before melt-blending, the masterbatch and PEEK powders were dried in an oven at 150 °C for 3 h under vacuum, and then they were ground by an agate mortar to get good dispersion.

Samples for mechanical and thermomechanical testing were prepared using a Haake MiniJet Pro injection molding machine (Thermal Scientific). The temperature of the cylinder and mold was set at 390 °C and 170 °C, respectively.

### 2.3. Characterization

ζ potential measurements were carried out by Zetasier Nano-ZS system (Malvern Instruments, Malvern, UK). The CNTs/MMT suspensions after sedimentation for 72 h were utilized. The samples were taken three times to average.

Transmission electron microscopy (TEM) was performed on a JEM-1230 (JEOL, Tokyo, Japan) at 90 kV. The TEM samples were obtained by ultra-thin sectioning (Lycra UC-7). The freezing temperature was −160 °C, and the slice thickness was about 100 nm.

Stress-strain experiment was carried out with AG-X Plus (Shimadzu, Kyoto, Japan) at room temperature. The crosshead speed was 10 mm/min. The dumbbell samples with a gauge length of 20 mm and width of 4 mm were used. At least five measurements were performed for each material.

The dynamic mechanical analysis of the nanocomposites was carried out by a DMA Q800 (TA instrument) in a single cantilever bending mode. The tests were performed at 1 Hz from 40 to 240 °C at a heating rate of 3 °C/min. An amplitude of 5 μm was applied. The dimension of the sample was 15 × 10 × 1 mm^3^.

The melting and crystallization behavior of the PEEK nanocomposites was probed using a differential scanning calorimetry (DSC, Q2000, TA instrument) under nitrogen. The sample weight was about 4.5 mg. For melting behavior, the DSC traces were recorded in heating at a rate of 10 °C/min for the injection molded samples. For crystallization behavior, the samples were maintained at 390 °C for 5 min and then cooled to 100 °C at various rates from 2.5 to 20 °C/min.

The spherulitic morphology of the PEEK nanocomposites was investigated using a polarized optical microscopy (BX61, Olympus, Tokyo, Japan) with a hot stage (THMS600, Linkam, Surrey, UK). The samples were melted at 400 °C for 5 min, then cooled to 320 °C at a rate of 50 °C/min for isothermal crystallization.

## 3. Results and Discussion

### 3.1. MMT-Assisted Dispersion of CNTs in Water

**Figure 2:**
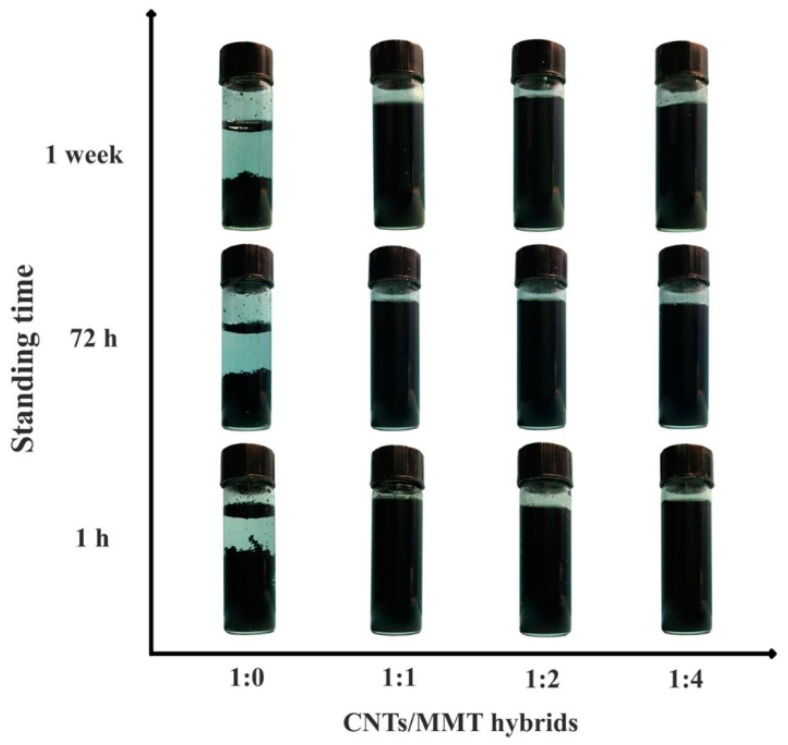
CNTs/MMT suspensions with various ratios.

Figure 2 shows the dispersion states of CNTs and CNTs/MMT suspension after standing for 24 h, 72 h, and 1 week. After standing for 24 h, transparent area at the top of the suspensions is about 1/3 of the total volume. As time continues, the transparent area becomes larger. The sedimentation of CNTs in water was also reported in the literature [34,35]. For CNTs/MMT hybrids with various ratios, the aqueous dispersions are still black after standing for 24 h, 72 h, or 1 week, respectively. This indicates that the incorporation of MMT promotes the dispersity of CNTs in water.

**Figure 3:**
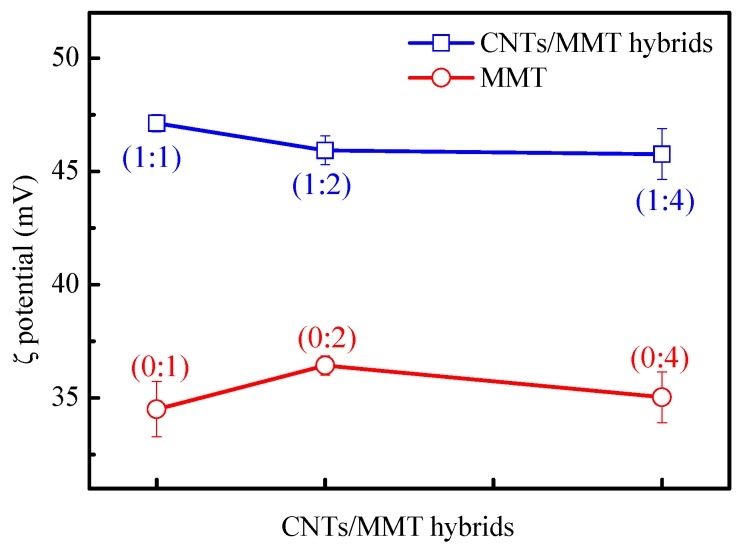
ζ potentials of MMT and CNTs/MMT suspensions with various ratios after standing for 72 h.

**Figure 4:**
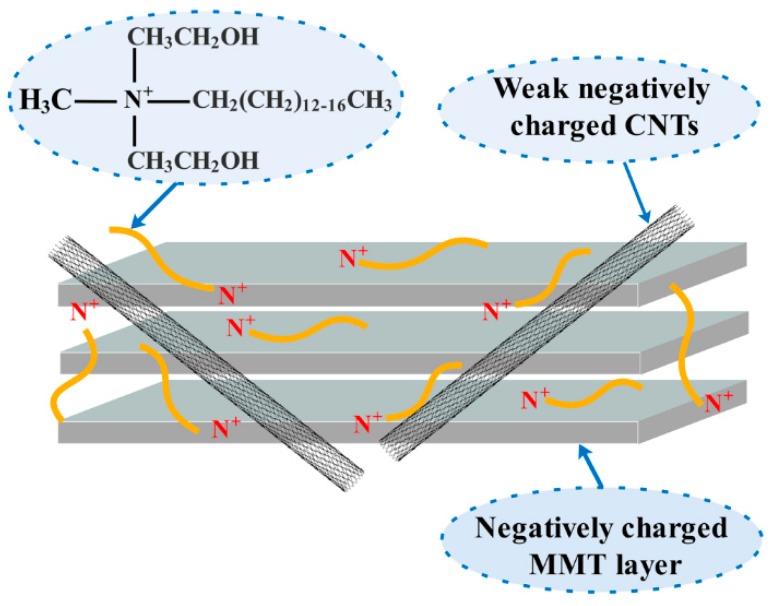
Schematic illustration of the interaction between CNTs and MMT.

ζ potential is useful in measuring the colloidal stability [36]. The ζ potentials of CNTs/MMT suspensions as a function of MMT content is shown in Figure 3. The ζ potential is around 35 mV for MMT with various concentrations, while its value increases to around 47 mV with the addition of CNTs, regardless of the ratio of CNTs/MMT. The increase in the value of ζ potential indicates better stability of CNTs/MMT suspension in water [37,38]. The stable dispersion of CNTs/MMT in water is probably due to a strong interaction between CNTs and MMT platelets, since the Na^+^ ion has a strong interaction with negatively charged CNTs [39,40]. Tang et al. suggested that the Na^+^ ion originating from MMT played the role of counterion between MMT sheets and CNTs [34]. Therefore, in our current work, the highly positively charged quaternary ammonium salt in organically modified MMT can also act as a counterion between MMT sheets and CNTs, leading to stable CNTs/MMT dispersion in water. A schematic illustration of the interaction between CNTs and MMT is shown in Figure 4.

### 3.2. Morphologies of PEEK Nanocomposites

**Figure 5:**
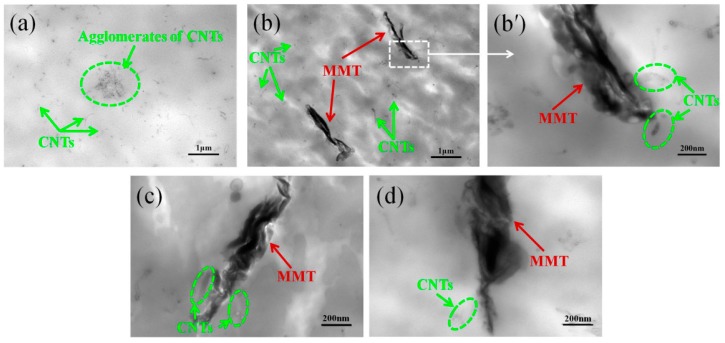
Representative TEM images of (**a**) PEEKC1 and (**b**) PEEKC1M1, (**b′**) is a partially enlarged image of (**b**), (**c**) PEEKC1M2, (**d**) PEEKC1M4.

The preformed structure of CNTs/MMT hybrids can be retained even after melt-processing, as confirmed from the TEM micrographs (Figure 5). CNTs tend to agglomerate in the PEEK matrix for PEEKC1 (Figure 5a), while good dispersion of CNTs is achieved in the matrix for PEEKC1M1 (Figure 5b). Moreover, a coating of CNTs around the MMT is observed (Figure 5b′,c,d). This can be ascribed to a strong interaction between CNTs and MMT. In addition, the TEM image indicates that the molecular chains of PEEK do not effectively diffuse into the MMT gallery during the melt-mixing process. A similar observation was reported in PEEK reinforced by organically modified MMT [12].

### 3.3. Tensile Behavior of PEEK Nanocomposites

**Figure 6:**
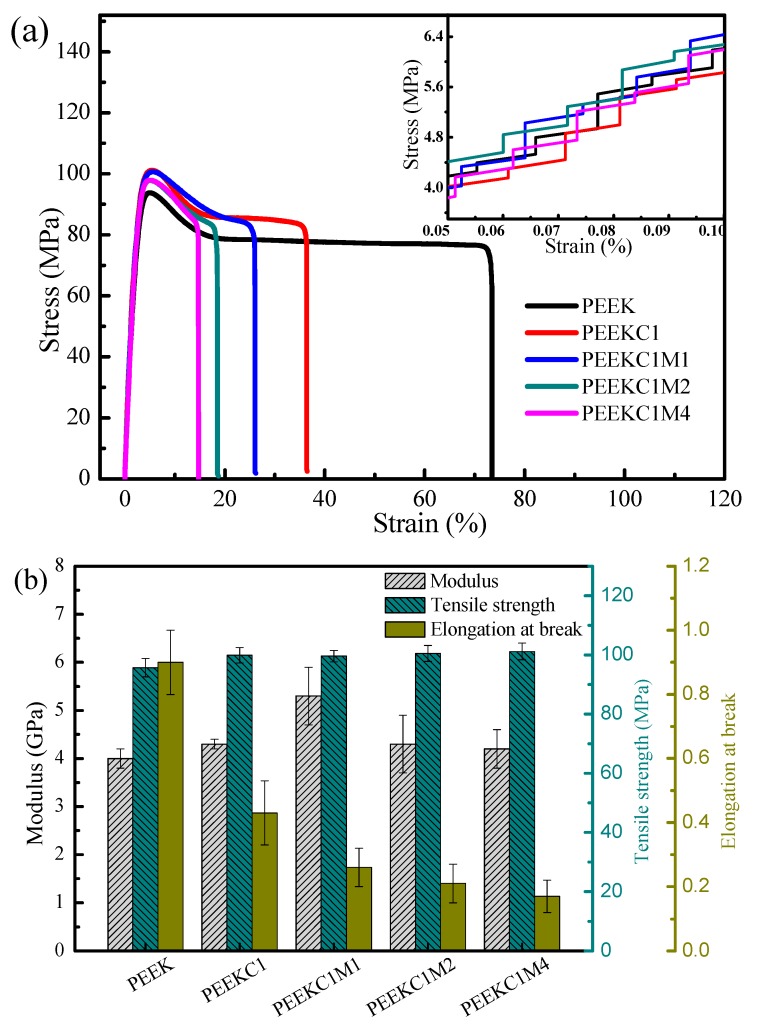
Typical engineering stress-strain curves of PEEK nanocomposites and the inset is the magnification of the linear region of the curve within which Young’s modulus is calculated (**a**), and the corresponding tensile properties (**b**).

Figure 6 shows the stress-strain curves of PEEK nanocomposites and the resultant modulus, tensile strength, and elongation at break. It is noteworthy that the modulus was evaluated from the linear portion of the curves (data at 0.05% to 0.1% of strain were used for linear regression). The modulus of PEEK is 4.0 GPa, while it is improved by 7.5% with the incorporation of 0.5 wt% CNTs in the PEEK matrix. Further addition of 0.5 wt% MMT leads to an increase in modulus by 23.3%, compared with PEEKC1. However, the modulus of PEEKC1M2 and PEEKC1M4 is slightly lower than that of PEEKC1. The modulus decrease at the increase of MMT is probably due to the fact that the molecular chains of PEEK do not effectively diffuse into the MMT gallery (Figure 5). In addition, crystallinity may also have an influence on the modulus of the nanocomposites. However, as is reported in Section 3.5, the crystallinity of PEEK nanocomposites is almost the same as that of neat PEEK. On the other hand, the tensile strength of the matrix increases marginally from 95.7 to 100.0 MPa with the incorporation of 0.5 wt% CNT, while it is almost not influenced by further addition of MMT. The elongation at break of PEEK nanocomposites decreases significantly with increasing the concentration of the nanofillers. Similar observations were reported for high-density polyethylene, thermoplastic polyurethane, and polypropylene (PP)/CNTs/MMT [28,41,42]. In brief summary, the incorporation of MMT as a second filler has a reinforcement effect on PEEK through improving the dispersion of CNTs in the matrix, while MMT itself has a negative effect on the mechanical properties of PEEK since the diffusion of PEEK into the MMT gallery is unsuccessful.

### 3.4. DMA Analysis of PEEK Nanocomposites

**Figure 7:**
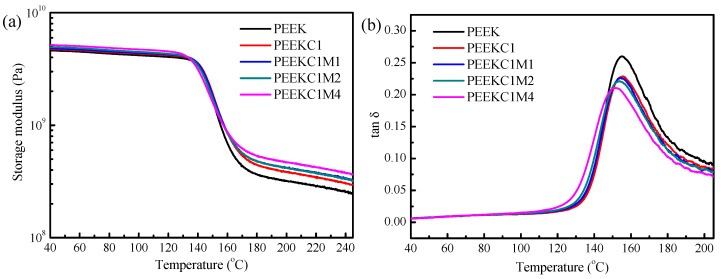
(**a**) Storage modulus *E*’ and (**b**) tan *δ* as a function of temperature, at the frequency of 1 Hz, for PEEK nanocomposites.

The DMA analysis of PEEK nanocomposites was also explored. The storage modulus *E*’ and tan δ curves as a function of temperature are presented in Figure 7. The *E*’ values at 40 and 240 °C (from Figure 7a), as well as the glass transition temperature (*T*_g_) taken from the tan δ curves (Figure 7b), are listed in Table 2. At 40 °C, the storage modulus of neat PEEK is 4.6 GPa. It increases marginally for PEEKC1 and PEEKC1M1. This trend is consistent with the improvement in tensile modulus of PEEK with the addition of CNTs and/or MMT (Figure 6). The marginal enhancement in the stiffness of the PEEK matrix can be ascribed to the high modulus of CNTs and/or MMT. It is noteworthy that the storage modulus of PEEKC1M2 and PEEKC1M4 is 5.0 and 5.2 GPa, respectively, which is higher than 4.7 GPa of PEEKC1. This trend is in contrast to the case of the tensile modulus (Figure 6). The inconsistency is possibly due to the different testing conditions in tensile testing and DMA. For DMA, a very small amplitude of 5 μm was used, while large deformation took place for tensile testing. Therefore, the stress concentration, which is induced by layered fillers, is more remarkable in tensile testing than that in DMA.

At 240 °C, neat PEEK presents a storage modulus of 255.1 MPa, which is much lower than that at 40 °C. This dramatic decrease in storage modulus corresponds to the material’s transition from a glassy to a rubbery state. Compared to neat PEEK, the storage modulus for PEEKC1 increases by 18.3%, and it increases by 32.1%, 30.9%, and 48.1% for PEEKC1M1, PEEKC1M2, and PEEKC1M4, respectively. On the other hand, the addition of MMT has a synergistic effect with CNTs in increasing the modulus of PEEK nanocomposites. A similar mechanical enhancement was also found in other polymer nanocomposites [27,43]. It is noteworthy that this dramatic improvement in storage modulus at the temperature as high as 240 °C is very important for the practical applications of PEEK, since it is intended to be used at high temperature as a high-performance engineering plastics.

As can be seen from Table 2, neat PEEK presents a *T*_g_ at 147.1 °C. The same value is observed for PEEKC1. However, further addition of 0.5, 1.0, and 2.0 wt% MMT decreases *T*_g_ of PEEK by 0.6, 1.7, and 4.7 °C, respectively. The decrease in *T*_g_ can be ascribed to the plasticization effect of the organic modifier in the modified MMT. It was reported that the *T*_g_ of PEEK with unmodified MMT increased 3 °C in comparison with that of neat PEEK, while the trend was opposite for PEEK with organoclay [12]. The formation of an intercalated structure is also possible, in which the tethered PEEK chains in the narrow spaces of MMT gain higher mobility [44].

### 3.5. Melting and Crystallization Behavior of PEEK Nanocomposites

**Figure 8:**
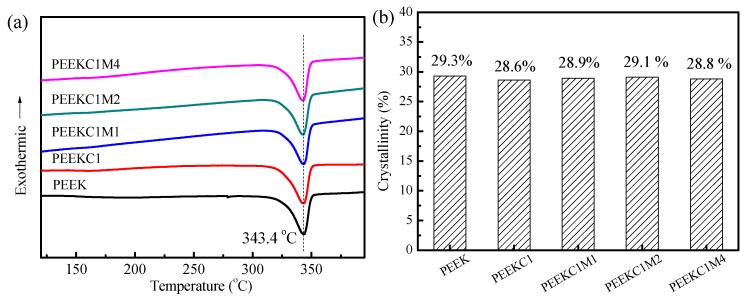
(**a**) DSC traces of PEEK nanocomposites recorded during heating at a rate of 10 °C/min for the injection molded samples, and (**b**) the resultant crystallinity.

To investigate the effect of CNTs and MMT on the melting behavior of PEEK, the DSC heating measurements were performed for the injection molded samples, as shown in Figure 8a. It is observed that the melting temperature is kept almost constant for neat PEEK and its nanocomposites. From the DSC data, the crystallinity of PEEK nanocomposites can be estimated as

*X* = Δ*H*_m_/(Δ*H*_m_^o^ · *ν*_m_) (1)

where Δ*H*_m_ is the melting enthalpy of the nanocomposites, Δ*H*_m_^o^ = 130 J/g is the value of an infinitely large crystal of PEEK [45], and *ν*_m_ is the polymer content. The resultant crystallinity of PEEK nanocomposites is shown in Figure 8b. The crystallinity of neat PEEK is 29.3%, and the value is almost the same as that of PEEK nanocomposites.

**Figure 9:**
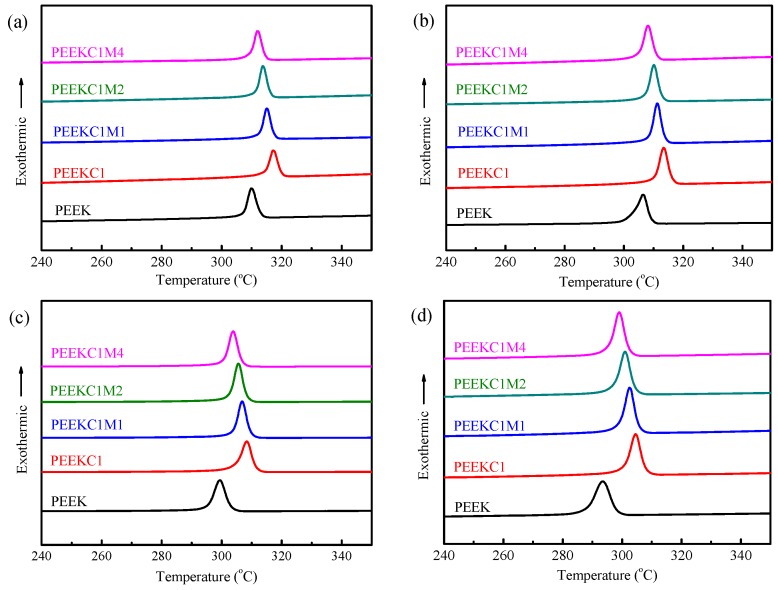
DSC traces of PEEK nanocomposites recorded during cooling process at (**a**) 2.5 °C/min, (**b**) 5 °C/min, (**c**) 10 °C/min, and (**d**) 20 °C/min after eliminating previous thermal histories.

**Figure 10:**
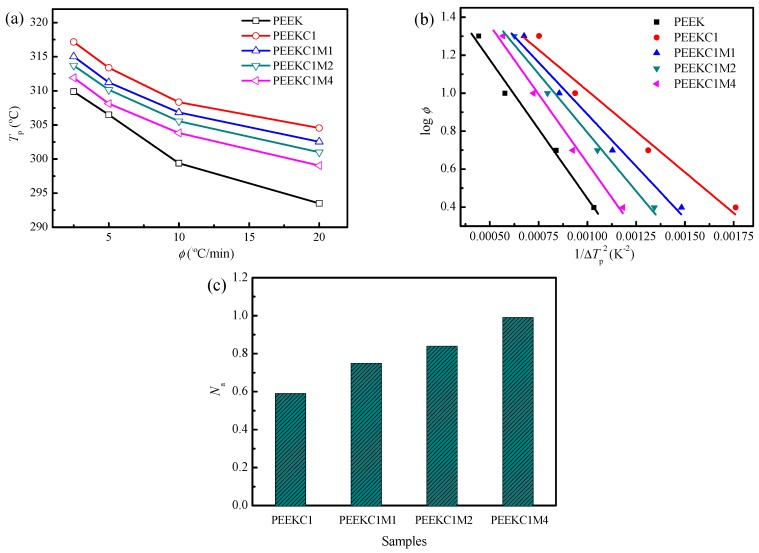
Correlation calculation of crystallization behavior: (**a**) *T*_p_ diagram as a function of cooling rate, (**b**) Dobreva plots of log *φ* versus 1/Δ*T*_p_^2^, and (**c**) variation of nucleation activity for PEEK nanocomposites.

As a semicrystalline polymer, the crystalline morphologies can have a profound impact on the properties of the material. Therefore, the nonisothermal crystallization behavior of PEEK nanocomposites was investigated by DSC, as shown in Figure 9. The peak temperature of crystallization (*T*_p_) as a function of the cooling rate is plotted at Figure 10a. As expected, the *T*_p_ of the crystallization exotherm decreases with an increasing cooling rate for each specimen. Similar behavior has been reported in PEEK and other polymer systems [46,47,48,49,50]. In addition, at each cooling rate, the addition of CNTs induces a higher *T*_p_. This indicates that CNTs act as nucleation agents in enhancing PEEK crystallization. Similar behavior was reported for PEEK/CNTs nanocomposites [19]. However, compared to that of PEEKC1, further incorporation of MMT results in a decrease of *T*_p_, and this trend is more significant with an increase in the concentration of MMT. For PEEK/MMT nanocomposites, it was found that MMT played a role in inhibiting PEEK crystallization [12]. The same conclusion is reasonable for PP/CNTs/MMT nanocomposites [28].

The Dobreva and Gutzow method was utilized to evaluate the nucleation activity of the fillers for polymers [51,52]. The nucleation activity can be evaluated from the equation:

*N*_a_ = B*/*B* (2)

where *B* is a parameter for the pristine polymer, while B* is for the polymer when it is filled. Both *B* and B* can be calculated experimentally from the slope of the following equation:

log *φ* = Constant − *B*( or B*)/(2.3 Δ*T*_p_^2^) (3)

where *φ* is the cooling rate, Δ*T*_p_ is the degree of supercooling (Δ*T*_p_ = *T*_m_ − *T*_c_). If the foreign substrate is extremely active, *N*_a_ approaches zero, while for inert particles it is unity. This approach has been successfully used to calculate the nucleating ability of mineral particles filled PP [53,54].

Plots of log *φ* versus 1/Δ*T*_p_^2^ for PEEK nanocomposites are shown in Figure 10b. The calculated values of *N*_a_ as a function of the materials are shown in Figure 10c. Clearly, the value of *N*_a_ for PEEKC1 is the lowest, indicating the highest nucleation of CNTs. The *N*_a_ value increases with the further addition of MMT, indicating that MMT inhibits the nucleation of PEEK.

**Figure 11:**
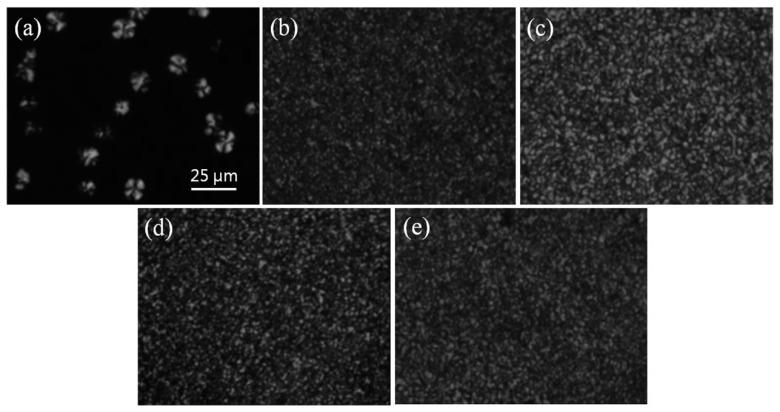
Spherulitic morphologies of (**a**) PEEK, (**b**) PEEKC1, (**c**) PEEKC1M1, (**d**) PEEKC1M2, and (**e**) PEEKC1M4 isothermally crystallized at 320 °C for 15 min from the melt.

Figure 11 presents the spherulitic morphology of neat PEEK and its nanocomposites crystallized isothermally at 320 °C. Obviously, the spherulites of PEEK nanocomposites are much smaller and denser in comparison with neat PEEK. This indicates that the nanofillers act as an effective nucleating agent of PEEK. However, the influence of the concentration of the nanofillers on the nucleating density of PEEK is not detected, due to the resolution limitations of optical microscopy.

## 4. Conclusions

In this work, nanostructured CNTs/MMT hybrids were fabricated to prepare a new class of PEEK nanocomposites and, meanwhile, to solve the agglomeration problem of CNTs. CNTs/MMT hybrids were achieved simply by mixing CNTs with MMT in water. The dispersion stability of CNTs was improved with the assistance of MMT in water, as characterized by sedimentation and zeta potential. PEEK nanocomposites were prepared via melt-mixing the PEEK/CNTs/MMT masterbatch with PEEK powder. The preformed structure of CNTs/MMT hybrids was maintained in PEEK nanocomposites as demonstrated by TEM. The mechanical and thermomechanical measurements revealed that CNTs together with MMT had a strong reinforcement effect on the PEEK matrix, especially at high temperature. DSC data revealed that CNTs accelerated the crystallization of the PEEK matrix while further addition of MMT played an opposite role. A similar trend was true for the nucleation activity of PEEK nanocomposites as evaluated by the Dobreva method. These results revealed that the addition of CNTs/MMT hybrids improved the material properties of PEEK, especially at high temperature.

## Figures and Tables

**Table 1:** The Designation and composition of the samples.

Sample Code	PEEK (wt%)	CNTs (wt%)	MMT (wt%)
PEEK	100	-	-
PEEKC1	99.5	0.5	-
PEEKC1M1	99.0	0.5	0.5
PEEKC1M2	98.5	0.5	1.0
PEEKC1M4	97.5	0.5	2.0

Poly(ether ether ketone), PEEK; multi-walled carbon nanotubes, CNTs; organically modified montmorillonite, MMT.

**Table 2:** Storage modulus *E*’ at 40 and 240 °C, and glass transition temperature *T*_g_ for PEEK nanocomposites obtained from DMA data in Figure 7.

Samples	*E*’_40 °C_ (GPa)	*E*’_240__°C_ (MPa)	*T*_g_ (°C)
PEEK	4.6	255.1	147.1
PEEKC1	4.7	301.9	147.1
PEEKC1M1	4.8	337.0	146.5
PEEKC1M2	5.0	334.0	145.4
PEEKC1M4	5.2	377.8	142.4
